# Low serum magnesium levels are associated with impaired peripheral nerve function in type 2 diabetic patients

**DOI:** 10.1038/srep32623

**Published:** 2016-09-07

**Authors:** Chen Chu, Weijing Zhao, Yinan Zhang, Lu Li, Jingyi Lu, Lan Jiang, Congrong Wang, Weiping Jia

**Affiliations:** 1 Shanghai Jiao Tong University Affiliated Sixth People’s Hospital, Department of Endocrinology and Metabolism, Shanghai Diabetes Institute, Shanghai Key Laboratory of Diabetes Mellitus, Shanghai Clinical Center for Diabetes, 600 Yishan Road, Shanghai 200233, People’s Republic of China; 2 Shanghai Jiao Tong University Affiliated Sixth People’s Hospital, Department of Endocrinology and Metabolism, Shanghai Diabetes Institute, 600 Yishan Road, Shanghai 200233, People’s Republic of China; 3 Shanghai Jiao Tong University Affiliated Sixth People’s Hospital, Center for Translational Medicine, Shanghai Key Laboratory of Diabetes Mellitus, The Metabolic Diseases Biobank, Shanghai, 200233, People’s Republic of China; 4 Shanghai Jiao Tong University Affiliated Sixth People’s Hospital, Department of Electrophysiology, Shanghai, 200233, People’s Republic of China

## Abstract

The aim of this study was to explore the relationship between serum magnesium and peripheral nerve function in patients with type 2 diabetes (T2DM). A total of 978 T2DM patients were included in the study. Patients were divided into tertiles according to serum magnesium concentration (low tertile: ≤0.85 mmol/L; medium tertile: 0.85 to 0.92 mmol/L; and high tertile: >0.92 mmol/L). All participants underwent nerve conduction (NC) studies. Composite z scores of conduction velocity, latency, and amplitude were constructed, respectively. The serum magnesium levels were significantly lower in patients with abnormal NC than in those with normal NC (0.87 [0.82, 0.92] vs. 0.88 [0.83, 0.93] mmol/L, *P* = 0.048). The composite z score of amplitude significantly increased with increasing tertiles of magnesium (−0.60 ± 0.02 vs. −0.57 ± 0.02 vs. −0.48 ± 0.03, *P* for trend = 0.001). After adjusting for all potential confounders, lower serum magnesium levels were still associated with lower composite z score of amplitude (*β* = 0.095, *P* = 0.014). In patients with T2DM, lower serum magnesium levels were significantly associated with lower composite z score of amplitude, indicating magnesium might affect peripheral nerve function through axonal degeneration.

Diabetes is becoming a major public health threat in China^1^. Despite improvements in the management of diabetes, diabetic peripheral neuropathy (DPN) has become the most commonly reported chronic diabetic complication, affecting up to half of diabetic patients^2^. DPN causes serious compilations, such as foot ulcers and gangrene, leading to lower limb amputation, all of which reduce the quality of life in diabetic patients^3^,^4^. Several pathogenic mechanisms have been reported to be involved in DPN, including microangiopathy, oxidative stress, inflammation, the polyol pathway, glycation, and ligand activation^5^,^6^,^7^,^8^. However, the underlying pathophysiology of DPN is complex and has not been fully elucidated.

Magnesium is the fourth most abundant cation in the human body. It acts as a co-factor for numerous enzymatic reactions and exerts important roles in many biological processes^9^,^10^. Recent studies have demonstrated that low serum magnesium is independently associated with an increased risk of type 2 diabetes (T2DM)^11^,^12^,^13^, cardiovascular disease^14^, and foot ulcers^15^. Furthermore, there is increasing evidence that hypomagnesemia is associated with microvascular complications of T2DM, such as nephropathy^16^,^17^,^18^ and retinopathy^19^,^20^. The relationship between serum magnesium and DPN remains unclear and controversial, with conflicting results observed regarding the effects of serum magnesium levels on nerve conduction (NC) and the presence of neuropathic pain^21^,^22^,^23^. Therefore, we designed this cross-sectional study to examine the association between serum magnesium levels and peripheral nerve function in patients with T2DM.

## Results

### Patient characteristics

The demographic and clinical characteristics of patients with normal and abnormal NC are illustrated in Table 1. Among the 978 patients, 746 (76.28%) had normal NC and 232 (23.72%) had abnormal NC. Abnormal NC was significantly associated with older age, longer diabetes duration (all *P* < 0.001). Besides, HbA1c, systolic blood pressure (SBP), urinary albumin excretion (UAE) and the percentage of anti-hypertensive therapy were significantly higher in the abnormal NC group (all *P* < 0.05). Interestingly, serum magnesium levels were significantly lower in the abnormal NC group (*P* = 0.048).

### Association of serum magnesium levels with NC parameters

Patients were further divided into tertiles according to serum magnesium levels (low tertile: ≤0.85 mmol/L; medium tertile: 0.85 to 0.92 mmol/L, and high tertile: >0.92 mmol/L). As presented in Table 2, patients in the high tertile of serum magnesium were older, had lower levels of body mass index (BMI), HbA1c, estimated glomerular filtration rate (eGFR) and UAE than those in the low and medium tertile (all *P* < 0.001). Besides, diastolic blood pressure (DBP), high-density lipoprotein cholesterol (HDL-c), triglycerides (TG), creatinine, and the percentage of anti-hypertensive therapy were significantly different among the 3 tertiles (all *P* < 0.05).

All individual nerve conduction studies (NCS) parameters were analyzed firstly. Significant differences were observed in amplitude for motor tibial (*P* = 0.046), sensory ulnar (*P* = 0.044) and sensory sural nerves (*P* = 0.012) among the three tertiles of serum magnesium (Table 2). However, there was no significant difference in the conduction velocity (CV) and latency for all tested nerves. In addition, there was no significant difference of CV, amplitude and latency for peroneal nerve among serum magnesium tertiles (Supplementary Table S1).

**Figure 1:**
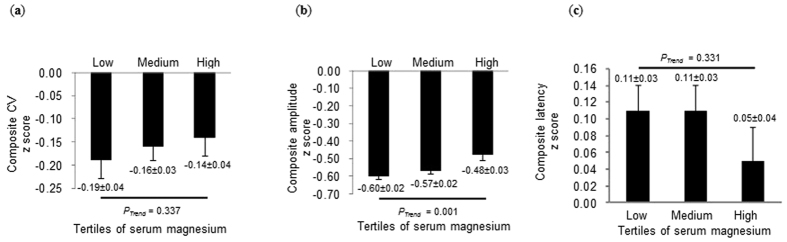
Composite z scores of nerve conduction parameters, according to tertiles of serum magnesium. Results were represented as mean ± standard error (s.e.m.). (**a**) Composite z score of conduction velocity (CV) across serum magnesium tertiles; (**b**) Composite z score of amplitude across serum magnesium tertiles; (**c**) Composite z score of latency across serum magnesium tertiles.

Next, composite z scores of CV, latency, and amplitude were calculated, respectively. The results showed that the composite z score of amplitude significantly increased with increasing tertiles of magnesium (−0.60 ± 0.02 vs. −0.57 ± 0.02 vs. −0.48 ± 0.03, *P* for trend = 0.001; Fig. 1b). In addition, post-hoc analysis using Bonferroni correction showed significant differences in the composite z score of amplitude between the medium and high tertile (*P* = 0.047) as well as between the low and high tertile (*P* = 0.002). Notably, the result regarding the composite z score of amplitude was consistent with that of amplitude analysis in individual nerves. However, no significant trends were observed among tertiles of magnesium with respect to the composite z scores of both CV (*P* for trend = 0.337) and latency (*P* for trend = 0.331).

Multiple linear regression analysis showed that serum magnesium was still positively correlated with the composite z score of amplitude (*β* = 0.102, *P* = 0.007; Table 3), after adjusting for age, sex, diabetes duration, HbA1c level, anti-hypertensive therapy, SBP, DBP, UAE (model 1). Given that serum magnesium has been shown to be negatively correlated with eGFR^14^,^24^ and our results were also in accordance with this observation (r = −0.19, P<0.001), eGFR was additionally included for adjustment (model 2). We found that the association of serum magnesium with the composite z score of amplitude (*β* = 0.095, *P* = 0.014) remained significant.

## Discussion

Serum magnesium has a close relationship with T2DM^11^,^12^. It has been shown that lower serum magnesium levels are significantly associated with increased risk of T2DM^13^ and with various complications of diabetes, including cardiovascular disease^14^, nephropathy^16^,^17^,^18^,^25^, retinopathy^19^,^20^, and foot ulcers^15^. However, data regarding the relationship between serum magnesium and DPN are limited and controversial. Previous research has shown that magnesium supplementation could improve both NC in type 1 diabetic patients^21^ and symptoms of neuropathy in streptozocin-induced diabetic (STZ-D) rats^22^. In contrast, Hyassat *et al*. found no association between hypomagnesemia and neuropathy when the diagnosis of neuropathy was based on neuropathic symptoms or the presence of an abnormality of NC^23^.

In this study, we performed NCS in all patients, which is an accurate, sensitive, and reproducible method for evaluating DPN^26^,^27^. Additionally, composite z scores of NCS parameters were constructed for six nerves, including motor nerves (median, ulnar, and tibial) and sensory nerves (median, ulnar, and sural)^28^. We found that serum magnesium levels were independently associated with the amplitude, but not with CV and latency. Interestingly, it is well established that low amplitude is an indicator of axonal degeneration, whereas decreased CV and prolonged latency are useful markers for demyelination^29^. It is plausible to postulate that serum magnesium levels might affect peripheral nerve function through axonal degeneration.

Magnesium exerts wide-range effects on many biological processes. First of all, increasing evidences indicated that magnesium can not only decrease tissue susceptibility to oxidative damage, but also has indirect antioxidant capacity^30^,^31^,^32^. Parvizi *et al*. found that treatment of STZ-D rats with MgSO4 can attenuate oxidative stress in the renal tissue as indicated by decreased levels of malondialdehyde^33^. Additionally, several studies have demonstrated that low serum magnesium concentration is closely associated with increased inflammation^34^,^35^. Furthermore, it has been reported that magnesium can increase intracellular inositol concentrations by enhancing the affinity of transport system for inositol, thus inhibiting further damage of the nervous system^32^,^36^. Although the multifactorial pathogenesis of DPN is still poorly understood, oxidative stress, inflammation and decreased intracellular inositol concentrations contribute to the progression of DPN^5^,^8^,^32^,^37^. In addition, myo-inositol may be at least partially responsible for the axonal degeneration^38^. Further studies are warranted to reveal the direct effects of low serum magnesium levels on axonal degeneration. Notably, in non-diabetic subjects, we did not observe any association between serum magnesium levels and the composite z scores of all NCS parameters, suggesting that magnesium will only affect NC function in diabetic patients.

In summary, our data showed that lower serum magnesium levels were significantly associated with lower composite z score of amplitude in patients with T2DM, indicating low serum magnesium levels might affect peripheral nerve function through axonal degeneration. Our finding suggested that low serum magnesium levels may underlie many of the pathophysiologic features of DPN. Further studies will provide a novel prospective strategy for DPN.

## Methods

### Study population

A total of 978 T2DM patients were recruited from the Shanghai Diabetes Institute Inpatient Database of Shanghai Jiao Tong University Affiliated Sixth People’s Hospital between April 2013 and August 2014. Inclusion criteria include the following: (1) established T2DM diagnosed according to the 1999 WHO definition (fasting plasma glucose ≥7.0 mmol/L and/or 2-h plasma glucose ≥11.1 mmol/L); (2) valid data of NCS. Exclusion criteria included: (1) missing data on age, sex, diabetes duration, and serum magnesium; (2) histories of persistent diarrhea or vomiting, progressive malignancy and severe renal insufficiency as defined by an eGFR <60 ml·min^−1^·1.73 m^−2^; (3) current use of high-dose (>40 mg/day) diuretics and/or magnesium supplementation; (4) histories of diseases that could affect NC (i.e., Guillain–Barre syndrome, chronic inflammatory demyelinating polyneuropathy, or carpal tunnel syndrome, etc.).

This study was conducted in accordance with the Declaration of Helsinki II and was approved by the institutional review board of Shanghai Jiao Tong University Affiliated Sixth People’s Hospital. Written informed consent was obtained from each participants.

### Clinical and laboratory analysis

Weight and height were measured with a standardized method by the same physician. BMI was calculated as the body weight (kg) divided by the square of the height (m). Information of alcohol use and smoking status were obtained using a standardized questionnaire. Participants consuming alcohol on a regular basis (≥30 g per week) for more than 1 year were defined as alcohol consumers^1^. Subjects who smoked at least one cigarette per day for over 6 months were defined as current smokers^39^. SBP and DBP were calculated as the average value of three measurements taken at 3 min intervals using a mercury sphygmomanometer. Information regarding anti-hypertensive therapy (i.e., Angiotensin-converting enzyme inhibitor, Angiotensin II receptor blocker, Calcium channel blocker, and β-blocker etc.) were obtained from all participants’ medical records.

Blood samples were collected after an overnight fast of 8–10 h. Glycated hemoglobin (HbA1c) was determined by high-performance liquid chromatography (HLC-723 G7, Tosoh, Japan). Serum magnesium levels were measured by the xylidyl blue method (Hitachi 7600 analyzer). Serum creatinine, total cholesterol (TC), HDL-c, low-density lipoprotein cholesterol (LDL-c), and TG were determined enzymatically (Hitachi 7600 chemical analyzer). The eGFR was calculated using the Modification of Diet in Renal Disease (MDRD) formula developed for the Chinese population: eGFR (ml·min^−1^ ·1.73 m^−2^) = 186 × (serum creatinine in μmol/L × 0.011)^−1.154^ × (age in years)^–0.203^ × (0.742 if female) × (1.233 if Chinese)^40^. UAE was obtained from at least two 24-h urine samples and determined as the mean of 24-h urine collections during the period of hospitalization.

### Nerve conduction studies

Peripheral nerve function was evaluated by NCS with the use of the EMG Myto, EBNeuro (ESAOTE, Florence, Italy), which has been described previously^41^. Briefly, NCS were performed by experienced electrophysiologists who were unaware of the laboratory results. During testing, temperatures were maintained at ≥35 °C for upper extremities and ≥32 °C for lower extremities. Motor nerve studies measured CV, compound muscle action potential (CMAP) amplitude, and distal latency in the median, ulnar, and tibial nerves. Sensory nerve studies contained the CV, sensory nerve action potential (SNAP) amplitude, and onset latency in the median, ulnar, and sural nerves. We then compared all obtained data with reference values from our laboratory. Throughout the study, all data were reviewed by the reading site to ensure overall quality. Abnormal NC was defined by abnormality of one or more parameters in two or more tested anatomical nerves^42^. Composite z scores of CV, amplitude, and latency were constructed as previously described^41^,^43^.

### F-wave analysis

Supramaximal percutaneous stimuli at the degree of 1 Hz were given to peroneal, tibial, median and ulnar nerves for ten times, respectively. F-wave was recorded at the extensor digitorum brevis, abductor hallucis muscle, abductor pollicis brevis, and abductor digiti minimi by means of the EMG Myto, EBNeuro (ESAOTE, Florence, Italy). Variables analyzed included F-wave minimum latency and persistence. F-wave persistence was defined as the number of the F-responses obtained with ten stimuli. The details of F-wave analysis were shown in Supplementary Table S2 and S3.

### Statistical analysis

Data were expressed as mean ± standard deviation (SD) for normal distribution variables or as median (25–75th percentiles) for skewed distribution variables. Categorical variables were presented as numbers (percentage). Differences between the normal NC group and abnormal NC group were evaluated using the Student’s t-test or Mann–Whitney U test for continuous variables and the chi-squared test for categorical variables. Differences among tertiles of serum magnesium were analysed by one-way ANOVA or the Kruskal-Wallis test, as well as the chi-squared test for categorical values. Bonferroni correction was used for the post-hoc analyses. Data with a skewed distribution (including serum magnesium, and eGFR) were logarithmically transformed before analysis. Multivariate linear regression analysis was used to assess the independent associations of serum magnesium with NCS parameters after adjusting for covariates. All *P* values were two-sided, and values of *P* < 0.05 were considered statistically significant. Statistical analyses were performed using SPSS version 19 (SPSS, Inc., Chicago, IL, USA).

## Additional Information

**How to cite this article**: Chu, C. *et al*. Low serum magnesium levels are associated with impaired peripheral nerve function in type 2 diabetic patients. *Sci. Rep.* **6**, 32623; doi: 10.1038/srep32623 (2016).

## Supplementary Material

Supplementary Information

## Figures and Tables

**Table 1:** Characteristics of patients in the study.

Variable	Total (N = 978)	Normal NC (N = 746)	Abnormal NC (N = 232)	*P* value
Age (years)	57.32 ± 12.77	56.43 ± 12.97	60.18 ± 11.65	<0.001
Male sex (n, %)	541 (55.32%)	410 (54.96%)	131 (56.47%)	0.687
Alcohol consumers (n, %)	177 (18.17%)	128 (17.23%)	49 (21.21%)	0.170
Current smokers (n, %)	229 (23.44%)	171 (22.95%)	58 (25.00%)	0.520
SBP (mmHg)	130.64 ± 15.27	129.79 ± 14.97	133.40 ± 15.96	0.002
DBP (mmHg)	79.17 ± 9.03	79.20 ± 9.06	79.07 ± 8.95	0.844
BMI (kg/m^2^)	25.21 (22.85, 27.73)	25.11 (22.84, 27.51)	25.39 (23.15, 28.65)	0.098
Diabetes duration (years)	8.00 (4.00, 14.00)	8.00 (3.00, 13.00)	10.00 (5.00, 15.75)	<0.001
HbA1c (%)	8.00 (7.10, 9.50)	7.80 (6.90, 9.30)	8.75 (7.60, 10.10)	<0.001
Total cholesterol (mmol/L)	4.59 (4.01, 5.32)	4.62 (4.04, 5.31)	4.50 (3.91, 5.36)	0.555
HDL-c (mmol/L)	1.02 (0.87, 1.23)	1.02 (0.87, 1.23)	1.02 (0.87, 1.21)	0.793
LDL-c(mmol/L)	2.80 (2.21, 3.38)	2.82 (2.26, 3.38)	2.74 (2.14, 3.39)	0.361
Triglyceride (mmol/L)	1.39 (1.01, 1.99)	1.38 (1.02, 1.97)	1.42 (1.01, 2.03)	0.863
Serum creatinine (μmol/L)	67.00 (55.00, 78.00)	67.00 (55.00, 78.00)	68.00 (56.50, 78.00)	0.505
eGFR (ml · min^−1^ · 1.73 m^−2^)	129.56 (111.69, 149.36)	130.01 (112.95, 149.75)	128.18 (106.21, 148.77)	0.284
UAE (mg/24h)	9.82 (6.27, 24.60)	9.32 (6.14, 20.47)	14.10 (7.08, 43.12)	<0.001
Anti-hypertensive therapy (n, %)	532 (54.40%)	385 (51.61%)	147 (63.36%)	0.002
Serum magnesium (mmol/L)	0.88 (0.83, 0.93)	0.88 (0.83, 0.93)	0.87 (0.82, 0.92)	0.048

Data were expressed as mean ± standard deviation (SD) for normal distribution variables or as median (25–75th percentiles) for skewed distribution variables. Categorical variables were expressed as numbers (percentage).

NC, nerve conduction; SBP, systolic blood pressure; DBP, diastolic blood pressure; BMI, body mass index; HbA1c, glycated hemoglobin; HDL-c, high density lipoprotein cholesterol; LDL-c, low density lipoprotein cholesterol; eGFR, estimated glomerular filtration rate; and UAE, urinary albumin excretion.

**Table 2:** Characteristics of patients and nerve conduction parameters, according to tertiles of serum magnesium.

Variable	Serum magnesium			*P* value
	Low tertile (N = 366)	Medium tertile (N = 340)	High tertile (N = 272)	
Serum magnesium (mmol/L)	≤0.85	0.85–0.92	>0.92	
Age (years)	54.98 ± 13.27	58.40 ± 12.69	59.13 ± 11.68	<0.001
Male sex (n, %)	190 (51.91%)	194 (57.06%)	157 (57.72%)	0.250
Alcohol consumers (n, %)	64 (17.53%)	65 (19.23%)	48 (17.71%)	0.822
Current smokers (n, %)	78 (21.31%)	90 (26.55%)	61 (22.43%)	0.234
SBP (mmHg)	130.89 ± 14.81	131.28 ± 15.36	129.50 ± 15.77	0.333
DBP (mmHg)	79.36 ± 8.47	80.20 ± 9.55	77.63 ± 8.94	0.002
BMI (kg/m^2^)	25.64 (23.24, 28.48)	25.20 (22.91, 27.66)	24.22 (22.41, 26.68)	<0.001
Diabetes duration (years)	9.00 (4.00, 14.00)	8.00 (4.00, 13.00)	8.00 (3.00, 14.00)	0.344
HbA1c (%)	8.60 (7.30, 10.00)	8.20 (7.20, 9.50)	7.40 (6.70, 8.70)	<0.001
Total cholesterol (mmol/L)	4.55 (4.00, 5.32)	4.65 (4.06, 5.41)	4.60 (3.99, 5.26)	0.495
HDL-c (mmol/L)	0.99 (0.86, 1.17)	1.02 (0.88, 1.23)	1.07 (0.89, 1.29)	0.006
LDL-c (mmol/L)	2.79 (2.14, 3.35)	2.87 (2.32, 3.41)	2.71 (2.17, 3.32)	0.221
Triglyceride (mmol/L)	1.50 (1.09, 2.21)	1.37 (1.00, 1.99)	1.29 (0.97, 1.82)	0.002
Serum creatinine (μmol/L)	64.00 (52.00, 77.00)	66.00 (56.00, 76.00)	70.00 (59.00, 81.00)	<0.001
eGFR (ml · min^−1^ · 1.73 m^−2^)	134.23 (114.51, 158.77)	131.49 (113.33, 148.26)	122.92 (104.84, 139.82)	<0.001
UAE (mg/ 24h)	13.06 (7.00, 34.40)	9.50 (6.29, 24.86)	7.50 (5.63, 14.95)	<0.001
Anti-hypertensive therapy (n, %)	218 (59.56%)	187 (55.00%)	127 (46.69%)	0.005
Motor median CV (m/s)	53.85 (51.20, 56.81)	53.75 (51.20, 56.63)	54.45 (51.33, 58.10)	0.105
Motor median amplitude (mv)	5.95 (4.28, 7.63)	5.80 (4.10, 7.50)	6.40 (4.50, 8.20)	0.130
Motor median latency (ms)	3.45 (3.10, 3.90)	3.50 (3.10, 3.80)	3.40 (3.00, 3.80)	0.201
Motor ulnar CV (m/s)	58.70 (54.30, 62.90)	58.30 (54.51, 62.18)	59.00 (54.79, 63.20)	0.412
Motor ulnar amplitude (mv)	4.40 (3.58, 5.70)	4.50 (3.50, 5.48)	4.75 (3.60, 6.00)	0.164
Motor ulnar latency (ms)	2.45 (2.20, 2.70)	2.40 (2.20, 2.70)	2.40 (2.20, 2.60)	0.125
Motor tibial CV (m/s)	42.90 (40.28, 47.10)	43.31 (40.70, 47.90)	43.78 (41.50, 47.10)	0.064
Motor tibial amplitude (mv)	6.10 (3.90, 8.13)	6.40 (4.13, 8.88)	6.60 (4.63, 9.38)	0.046
Motor tibial latency (ms)	3.60 (3.20, 4.10)	3.50 (3.20, 4.00)	3.50 (3.10, 4.10)	0.517
Sensory median CV (m/s)	56.00 (50.78, 61.93)	56.00 (50.08, 61.13)	55.20 (51.63, 60.90)	0.836
Sensory median amplitude (mv)	9.30 (6.10, 13.00)	9.00 (5.75, 13.93)	10.00 (6.40, 15.00)	0.124
Sensory median latency (ms)	2.50 (2.30, 2.80)	2.50 (2.20, 2.80)	2.50 (2.30, 2.70)	0.791
Sensory ulnar CV (m/s)	58.00 (52.40, 62.50)	57.11 (52.30, 61.90)	58.00 (52.20, 62.50)	0.926
Sensory ulnar amplitude (mv)	7.95 (5.20, 10.50)	8.30 (5.90, 11.60)	8.35 (5.80, 11.00)	0.044
Sensory ulnar latency (ms)	2.10 (1.90, 2.30)	2.10 (1.92, 2.40)	2.10 (1.90, 2.38)	0.792
Sensory sural CV (m/s)	46.20 (42.38, 51.55)	46.95 (42.90, 52.08)	46.85 (42.90, 50.70)	0.745
Sensory sural amplitude (mv)	10.00 (5.88, 15.00)	10.20 (6.60, 16.00)	11.00 (7.03, 18.80)	0.012
Sensory sural latency (ms)	1.90 (1.50, 2.40)	2.00 (1.60, 2.50)	1.90 (1.50, 2.40)	0.183

Data were expressed as mean ± standard deviation (SD) for normal distribution variables or as median (25–75th percentiles) for skewed distribution variables. Categorical variables were expressed as numbers (percentage).

SBP, systolic blood pressure; DBP, diastolic blood pressure; BMI, body mass index; HbA1c, glycated hemoglobin; HDL-c, high density lipoprotein cholesterol; LDL-c, low density lipoprotein cholesterol; eGFR, estimated glomerular filtration rate; UAE, urinary albumin excretion; and CV, conduction velocity.

**Table 3:** Association of serum magnesium levels with nerve conduction parameters after adjustments.

Variable	Serum magnesium	
	*β*	*P* value
Model 1		
Composite z score of CV	0.010	0.792
Composite z score of amplitude	0.102	0.007
Composite z score of latency	−0.025	0.483
Model 2		
Composite z score of CV	0.004	0.918
Composite z score of amplitude	0.095	0.014
Composite z score of latency	−0.023	0.526

CV, conduction velocity.

Model 1: adjusted for age, sex, diabetes duration, HbA1c, anti-hypertensive therapy, systolic blood pressure, diastolic blood pressure, urinary albumin excretion.

Model 2: Model 1 + estimated glomerular filtration rate.

## References

[b1] Yang W. . Prevalence of diabetes among men and women in China. N Engl J Med 362, 1090–1101, 10.1056/NEJMoa0908292 (2010). 20335585

[b2] Tesfaye S. . Diabetic neuropathies: update on definitions, diagnostic criteria, estimation of severity, and treatments. Diabetes Care 33, 2285–2293, 10.2337/dc10-1303 (2010). 20876709 PMC2945176

[b3] Malik R. A. Which test for diagnosing early human diabetic neuropathy? Diabetes 63, 2206–2208, 10.2337/db14-0492 (2014). 24962918

[b4] Boulton A. J., Vileikyte L., Ragnarson-Tennvall G. & Apelqvist J. The global burden of diabetic foot disease. Lancet 366, 1719–1724, 10.1016/s0140-6736(05)67698-2 (2005). 16291066

[b5] Pasnoor M., Dimachkie M. M., Kluding P. & Barohn R. J. Diabetic neuropathy part 1: overview and symmetric phenotypes. Neurol Clin 31, 425–445, 10.1016/j.ncl.2013.02.004 (2013). 23642717 PMC4090918

[b6] Toth C. . Receptor for advanced glycation end products (RAGEs) and experimental diabetic neuropathy. Diabetes 57, 1002–1017, 10.2337/db07-0339 (2008). 18039814

[b7] Zochodne D. W. Diabetic polyneuropathy: an update. Curr Opin Neurol 21, 527–533, 10.1097/WCO.0b013e32830b84cb (2008). 18769245

[b8] Vincent A. M., Russell J. W., Low P. & Feldman E. L. Oxidative stress in the pathogenesis of diabetic neuropathy. Endocr Rev 25, 612–628, 10.1210/er.2003-0019 (2004). 15294884

[b9] Pham P. C., Pham P. M., Pham S. V., Miller J. M. & Pham P. T. Hypomagnesemia in patients with type 2 diabetes. Clin J Am Soc Nephrol 2, 366–373, 10.2215/cjn.02960906 (2007). 17699436

[b10] Saris N. E., Mervaala E., Karppanen H., Khawaja J. A. & Lewenstam A. Magnesium. An update on physiological, clinical and analytical aspects. Clin Chim Acta 294, 1–26 (2000). 10727669 10.1016/s0009-8981(99)00258-2

[b11] Dong J. Y., Xun P., He K. & Qin L. Q. Magnesium intake and risk of type 2 diabetes: meta-analysis of prospective cohort studies. Diabetes Care 34, 2116–2122, 10.2337/dc11-0518 (2011). 21868780 PMC3161260

[b12] Sales C. H. & Pedrosa Lde F. Magnesium and diabetes mellitus: their relation. Clin Nutr 25, 554–562, 10.1016/j.clnu.2006.03.003 (2006). 16690176

[b13] Fang C. . Association of Serum Magnesium Level with Odds of Prediabetes and Diabetes in a Southern Chinese Population: a Prospective Nested Case-Control Study. Biol Trace Elem Res 172, 307–314, 10.1007/s12011-015-0594-y (2016). 26706038

[b14] Wang S. . Serum electrolyte levels in relation to macrovascular complications in Chinese patients with diabetes mellitus. Cardiovasc Diabetol 12, 146, 10.1186/1475-2840-12-146 (2013). 24112518 PMC3852555

[b15] Rodriguez-Moran M. & Guerrero-Romero F. Low serum magnesium levels and foot ulcers in subjects with type 2 diabetes. Arch Med Res 32, 300–303 (2001). 11440788 10.1016/s0188-4409(01)00298-3

[b16] Corsonello A. . Serum ionized magnesium levels in type 2 diabetic patients with microalbuminuria or clinical proteinuria. Am J Nephrol 20, 187–192, doi: 13582 (2000). 10878399 10.1159/000013582

[b17] Pham P. C. . Lower serum magnesium levels are associated with more rapid decline of renal function in patients with diabetes mellitus type 2. Clin Nephrol 63, 429–436 (2005). 15960144 10.5414/cnp63429

[b18] Pham P. C. . The link between lower serum magnesium and kidney function in patients with diabetes mellitus Type 2 deserves a closer look. Clin Nephrol 71, 375–379 (2009). 19356369 10.5414/cnp71375

[b19] de Valk H. W., Hardus P. L., van Rijn H. J. & Erkelens D. W. Plasma magnesium concentration and progression of retinopathy. Diabetes Care 22, 864–865 (1999). 10332706 10.2337/diacare.22.5.864

[b20] Kundu D. . Serum magnesium levels in patients with diabetic retinopathy. J Nat Sci Biol Med 4, 113–116, 10.4103/0976-9668.107270 (2013). 23633845 PMC3633259

[b21] Engelen W., Bouten A., De Leeuw I. & De Block C. Are low magnesium levels in type 1 diabetes associated with electromyographical signs of polyneuropathy? Magnes Res 13, 197–203 (2000). 11008927

[b22] Rondon L. J. . Magnesium attenuates chronic hypersensitivity and spinal cord NMDA receptor phosphorylation in a rat model of diabetic neuropathic pain. J Physiol 588, 4205–4215, 10.1113/jphysiol.2010.197004 (2010). 20837644 PMC3002451

[b23] Hyassat D., Al Sitri E., Batieha A., El-Khateeb M. & Ajlouni K. Prevalence of Hypomagnesaemia among Obese Type 2 Diabetic Patients Attending the National Center for Diabetes, Endocrinology and Genetics (NCDEG). Int J Endocrinol Metab 12, e17796, 10.5812/ijem.17796 (2014). 25237327 PMC4166041

[b24] Pham P. C., Pham P. M. & Pham P. T. Patients with diabetes mellitus type 2 and hypomagnesemia may have enhanced glomerular filtration via hypocalcemia. Clin Nephrol 78, 442–448, 10.5414/cn107525 (2012). 23073059

[b25] Sakaguchi Y. . Hypomagnesemia in type 2 diabetic nephropathy: a novel predictor of end-stage renal disease. Diabetes Care 35, 1591–1597, 10.2337/dc12-0226 (2012). 22498805 PMC3379604

[b26] Weisman A. . Identification and prediction of diabetic sensorimotor polyneuropathy using individual and simple combinations of nerve conduction study parameters. PLoS One 8, e58783, 10.1371/journal.pone.0058783 (2013). 23533591 PMC3606395

[b27] Perkins B. A. & Bril V. Diabetic neuropathy: a review emphasizing diagnostic methods. Clin Neurophysiol 114, 1167–1175 (2003). 12842711 10.1016/s1388-2457(03)00025-7

[b28] Dyck P. J. . Individual attributes versus composite scores of nerve conduction abnormality: sensitivity, reproducibility, and concordance with impairment. Muscle Nerve 27, 202–210, 10.1002/mus.10320 (2003). 12548528

[b29] Chung T., Prasad K. & Lloyd T. E. Peripheral neuropathy: clinical and electrophysiological considerations. Neuroimaging Clin N Am 24, 49–65, 10.1016/j.nic.2013.03.023 (2014). 24210312 PMC4329247

[b30] Bonnefont-Rousselot D. The role of antioxidant micronutrients in the prevention of diabetic complications. Treat Endocrinol 3, 41–52 (2004). 15743112 10.2165/00024677-200403010-00005

[b31] Mazur A. . Magnesium and the inflammatory response: potential physiopathological implications. Arch Biochem Biophys 458, 48–56, 10.1016/j.abb.2006.03.031 (2007). 16712775

[b32] Hasanein P. . Oral magnesium administration prevents thermal hyperalgesia induced by diabetes in rats. Diabetes Res Clin Pract 73, 17–22, 10.1016/j.diabres.2005.12.004 (2006). 16417942

[b33] Parvizi M. R. . Protective effect of magnesium on renal function in STZ-induced diabetic rats. J Diabetes Metab Disord 13, 84, 10.1186/s40200-014-0084-3 (2014). 25197628 PMC4156611

[b34] Nielsen F. H. Magnesium, inflammation, and obesity in chronic disease. Nutr Rev 68, 333–340, 10.1111/j.1753-4887.2010.00293.x (2010). 20536778

[b35] Guerrero-Romero F. & Rodriguez-Moran M. Relationship between serum magnesium levels and C-reactive protein concentration, in non-diabetic, non-hypertensive obese subjects. Int J Obes Relat Metab Disord 26, 469–474 (2002). 12075573 10.1038/sj.ijo.0801954

[b36] Grafton G., Bunce C. M., Sheppard M. C., Brown G. & Baxter M. A. Effect of Mg2+ on Na(+)-dependent inositol transport. Role for Mg2+ in etiology of diabetic complications. Diabetes 41, 35–39 (1992). 1727737 10.2337/diab.41.1.35

[b37] Sandireddy R., Yerra V. G., Areti A., Komirishetty P. & Kumar A. Neuroinflammation and oxidative stress in diabetic neuropathy: futuristic strategies based on these targets. 2014, 674987, 10.1155/2014/674987 (2014). PMC4021687 24883061

[b38] Mayer J. H. & Tomlinson D. R. Prevention of defects of axonal transport and nerve conduction velocity by oral administration of myo-inositol or an aldose reductase inhibitor in streptozotocin-diabetic rats. Diabetologia 25, 433–438 (1983). 6197336 10.1007/BF00282524

[b39] Shi L. . Physical activity, smoking, and alcohol consumption in association with incidence of type 2 diabetes among middle-aged and elderly Chinese men. PLoS One 8, e77919, 10.1371/journal.pone.0077919 (2013). 24223743 PMC3817165

[b40] Ma Y. C. . Modified glomerular filtration rate estimating equation for Chinese patients with chronic kidney disease. J Am Soc Nephrol 17, 2937–2944, 10.1681/asn.2006040368 (2006). 16988059

[b41] Li L. . Serum albumin is associated with peripheral nerve function in patients with type 2 diabetes. Endocrine 50, 397–404, 10.1007/s12020-015-0588-8 (2015). 25860885

[b42] Dyck P. J., Carter R. E. & Litchy W. J. Modeling nerve conduction criteria for diagnosis of diabetic polyneuropathy. Muscle Nerve 44, 340–345, 10.1002/mus.22074 (2011). 21996793 PMC3193597

[b43] Pourhamidi K., Dahlin L. B., Boman K. & Rolandsson O. Heat shock protein 27 is associated with better nerve function and fewer signs of neuropathy. Diabetologia 54, 3143–3149, 10.1007/s00125-011-2303-5 (2011). 21909836

