# Supplementary material for: Low serum magnesium levels are associated with impaired peripheral nerve function in type 2 diabetic patients
Source: Sci Rep. 2016 Sep 7;6:32623. doi: 10.1038/srep32623 (PMC5013481; doi:10.1038/srep32623)
Supplement: Supplementary Information [file srep32623-s1.pdf]

Low serum magnesium levels are associated with impaired peripheral nerve function in type 2 diabetic patients

Chen Chu[1,+], Weijing Zhao[2,+], Yinan Zhang[3,+], Lu Li[1], Jingyi Lu[1], Lan Jiang[4], Congrong Wang[1,*] & Weiping Jia[1,*]

[1] Shanghai Jiao Tong University Affiliated Sixth People's Hospital, Department of Endocrinology and Metabolism, Shanghai Diabetes Institute, Shanghai Key Laboratory of Diabetes Mellitus, Shanghai Clinical Center for Diabetes, 600 Yishan Road, Shanghai 200233, People's Republic of China

[2] Shanghai Jiao Tong University Affiliated Sixth People's Hospital, Department of Endocrinology and Metabolism, Shanghai Diabetes Institute, 600 Yishan Road, Shanghai 200233, People's Republic of China

[3] Shanghai Jiao Tong University Affiliated Sixth People's Hospital, Center for Translational Medicine, Shanghai Key Laboratory of Diabetes Mellitus, The Metabolic Diseases Biobank, Shanghai, 200233, People's Republic of China

[4] Shanghai Jiao Tong University Affiliated Sixth People's Hospital, Department of Electrophysiology, Shanghai, 200233, People's Republic of China

* Correspondence and requests for materials should be addressed to C.W. (email: crwang@sjtu.edu.cn) or W.J. (email: wpjia@sjtu.edu.cn).

[+] These authors contributed equally to this work.

Supplementary information

Supplementary tables

Supplementary Table S1. Characteristics of nerve conduction parameters of peroneal nerve, according to tertiles of serum magnesium.

Variable	Serum magnesium			*P* value
	Low tertile (N = 358)	Medium tertile (N = 336)	High tertile (N = 269)	
Serum magnesium (mmol/L)	≤0.85	0.85-0.92	> 0.92	
Motor peroneal CV (m/s)	43.25 (41.08, 46.50)	43.50 (41.00, 46.20)	44.20 (41.50, 47.10)	0.053
Motor peroneal amplitude (mv)	3.90 (2.50, 5.60)	4.20 (2.30, 5.60)	4.00 (2.70, 6.15)	0.307
Motor peroneal latency (ms)	3.30 (3.00, 3.80)	3.20 (2.90, 3.70)	3.20 (2.90, 3.80)	0.075

The data were shown as median (25th-75th percentiles).

CV, conduction velocity.

Supplementary Table S2. Characteristics of F-wave parameters, according to groups of nerve function.

Variable	Total (N = 895)	Normal NC (N = 676)	Abnormal NC (N = 219)	*P* value
Minimum F-wave latencies (ms)				
Motor peroneal	48.50 (45.20, 53.80)	48.10 (44.83, 52.70)	50.10 (46.00, 56.50)	0.001
Motor tibial	48.30(45.20, 51.20)	47.70 (44.80,50.58)	50.10 (46.80, 54.10)	<0.001
Motor median	25.60 (24.10, 27.30)	25.40 (23.90, 27.10)	26.30 (24.80, 28.30)	<0.001
Motor ulnar	25.90 (24.40, 27.50)	25.80 (24.30, 27.30)	26.40 (24.90, 28.70)	<0.001
F-wave persistence (%)				
Motor peroneal	58.80 ±35.95	60.09 ±35.47	54.82 ±37.23	0.060
Motor tibial	95.30 ±16.01	96.02 ±13.78	93.04 ±21.46	0.057
Motor median	89.62 ±18.51	90.12 ±18.32	88.05 ±19.06	0.153
Motor ulnar	95.89 ±11.50	96.24 ±10.80	94.81 ±13.45	0.159

Data were expressed as mean ± standard deviation (SD) for normal distribution variables or as median (25th-75th percentiles) for skewed distribution variables.
NC, nerve conduction.

Supplementary Table S3. Characteristics of F-wave parameters, according to tertiles of serum magnesium.

Variable	Serum magnesium			*P* value
	Low tertile (N=337)	Medium tertile (N=307)	High tertile (N=251)	
Minimum F-wave latencies (ms)				
Motor peroneal	49.30 (45.40, 54.80)	48.80 (45.30, 53.40)	47.50 (43.60, 52.30)	0.015
Motor tibial	48.60 (45.30, 51.40)	48.90 (46.00, 51.50)	47.20 (44.40, 50.70)	0.003
Motor median	25.80 (24.20, 27.75)	25.60 (24.20, 27.30)	25.40 (24.00, 27.10)	0.241
Motor ulnar	26.20 (24.40, 28.00)	26.00 (24.40, 27.40)	25.50 (24.10, 27.30)	0.054
F-wave persistence (%)				
Motor peroneal	57.66 ±37.07	60.20 ±35.65	58.64 ±34.86	0.668
Motor tibial	95.67 ±15.11	94.59 ±17.82	95.68 ±14.83	0.631
Motor median	90.24 ±17.81	88.86 ±18.74	89.72 ±19.19	0.637
Motor ulnar	95.83 ±12.06	94.93 ±12.37	97.16 ±9.37	0.075

Data were expressed as mean ± standard deviation (SD) for normal distribution variables or as median (25th-75th percentiles) for skewed distribution variables.
